# Subcutaneous Emphysema and Pneumomediastinum Triggered by Nasotracheal Intubation: A Case Report

**DOI:** 10.7759/cureus.95653

**Published:** 2025-10-29

**Authors:** Yusuke Kurosawa, Karen Gomi, Akinori Moroi, Kunio Yoshizawa, Koichiro Ueki

**Affiliations:** 1 Department of Oral and Maxillofacial Surgery, Division of Medicine, Interdisciplinary Graduate School, University of Yamanashi, Chuo, JPN

**Keywords:** airway management, emphysema, intratracheal, intubation, oral surgical procedures, pneumomediastinum, subcutaneous

## Abstract

Nasotracheal intubation (NTI) is frequently chosen in oral and maxillofacial surgery to provide unobstructive access to the surgical field. Despite being rare, subcutaneous emphysema (SE) and pneumomediastinum (PM) caused by nasopharyngeal wall injury are serious complications. In this report, we present the case of a 74-year-old woman who developed right cervical SE and PM during anesthesia induction for oral surgery under general anesthesia associated with nasopharyngeal wall injury due to NTI. She had no remarkable medical history or abnormal preoperative findings. During intubation, swelling and crepitus of the right neck were observed; postoperative imaging confirmed extensive SE and PM. Conservative management with antibiotics was initiated immediately post-surgery, leading to the gradual resolution of symptoms. The patient was discharged on postoperative day 5. Although SE and PM secondary to pharyngeal trauma during tracheal intubation under general anesthesia are rare, careful selection of the airway management method, along with appropriate postoperative monitoring and early intervention, is essential in oral and maxillofacial surgery.

## Introduction

Nasotracheal intubation (NTI) is a standard technique used to secure the airway during oral and maxillofacial surgery. It is performed by advancing an endotracheal tube through the nostril into the trachea, typically under direct or fiberoptic visualization, to maintain a stable airway while keeping the oral cavity unobstructed for intraoral procedures. Although minor complications such as epistaxis and turbinate trauma are relatively common, particularly with oversized tubes or repeated blind attempts, more serious injuries may occur because the tube must traverse the nasal cavity, nasopharynx, and upper pharynx, where mucosal or submucosal damage can lead to subcutaneous emphysema (SE) and pneumomediastinum (PM) [1-3]. Although PM often resolves spontaneously, in advanced cases, it can cause circulatory failure due to impaired venous return and respiratory compromise, making early recognition and appropriate risk stratification crucial [4-6]. In particular, the posterior wall of the nasopharynx is anatomically thin and highly mobile, and its fragility increases with age, lowering the threshold for injury. Once damage occurs, air may spread continuously from the retropharyngeal space through the danger space into the mediastinum [5-7]. Herein, we report the case of a 74-year-old woman who developed right cervical SE and PM following nasopharyngeal wall injury during NTI for palatal torus reduction under general anesthesia.

## Case presentation

A 74-year-old woman presented to our department in October 2024 with a chief complaint of discomfort in the maxilla. Her medical history included nontuberculous mycobacterial infection, and her family history was unremarkable. She had persistent pain during mastication and difficulty with denture adaptation. Based on clinical findings, a diagnosis of palatal torus of the maxilla was made, and, considering the patient's strong preference, surgical removal of the torus under general anesthesia was planned.

General anesthesia was induced with total intravenous anesthesia (TIVA). NTI was performed by an attending anesthesiologist with over 10 years of clinical experience in airway management. A video laryngoscope-assisted nasotracheal technique was used under direct visualization (guided, not blind). For clarity, an "attempt" was defined a priori as a single passage of the tube from the nostril to the oropharynx under continuous direct laryngoscopic visualization. A lubricated cuffed oral/nasal tracheal tube (internal diameter (ID): 6.5 mm; Covidien™ Shiley™ oral/nasal tracheal tube, cuffed) was inserted through the right nasal cavity; however, marked resistance was encountered at the level of the inferior turbinate due to intranasal narrowing. Two passes were undertaken with the 6.5 mm tube. Upon encountering resistance, the tube was gently rotated to lateralize the bevel and subsequently withdrawn. A smaller tube (ID: 6 mm) was then introduced for two further passes; despite bevel-lateralizing rotation during the second pass, advancement beyond the nasal passage could not be achieved. Magill forceps were not used. Consequently, the approach was promptly converted to orotracheal intubation, which secured the airway and adequate ventilation.

**Figure 1:**
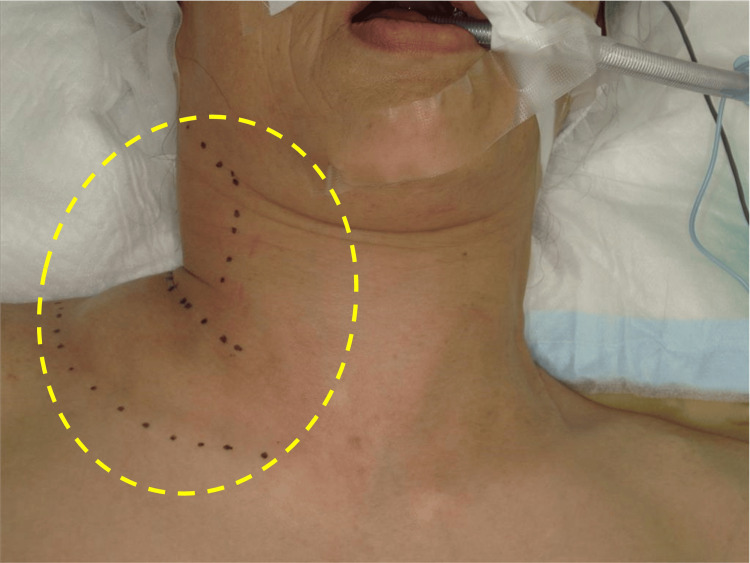
Clinical photograph of the right cervical region immediately following intubation demonstrating noticeable swelling

During intubation, swelling and crepitus of the right cervical region were observed (Figure 1), raising the suspicion of intubation-associated SE. Nevertheless, oxygenation and hemodynamic parameters remained stable, and the planned surgery was continued. The procedure lasted 35 minutes with 10 mL of blood loss and was completed uneventfully.

**Figure 2:**
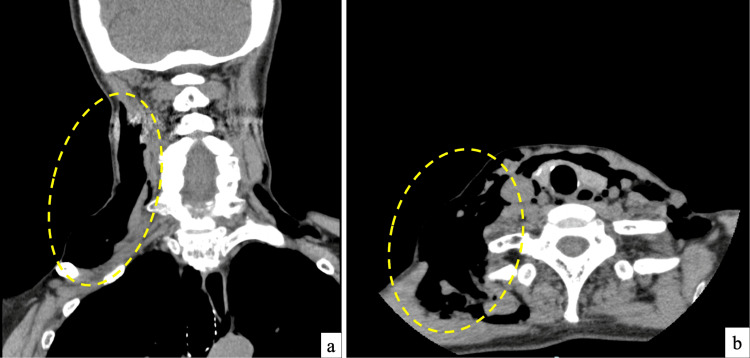
(a, b) Postoperative CT images demonstrating the presence of free air in the right cervical region CT: computed tomography

**Figure 3:**
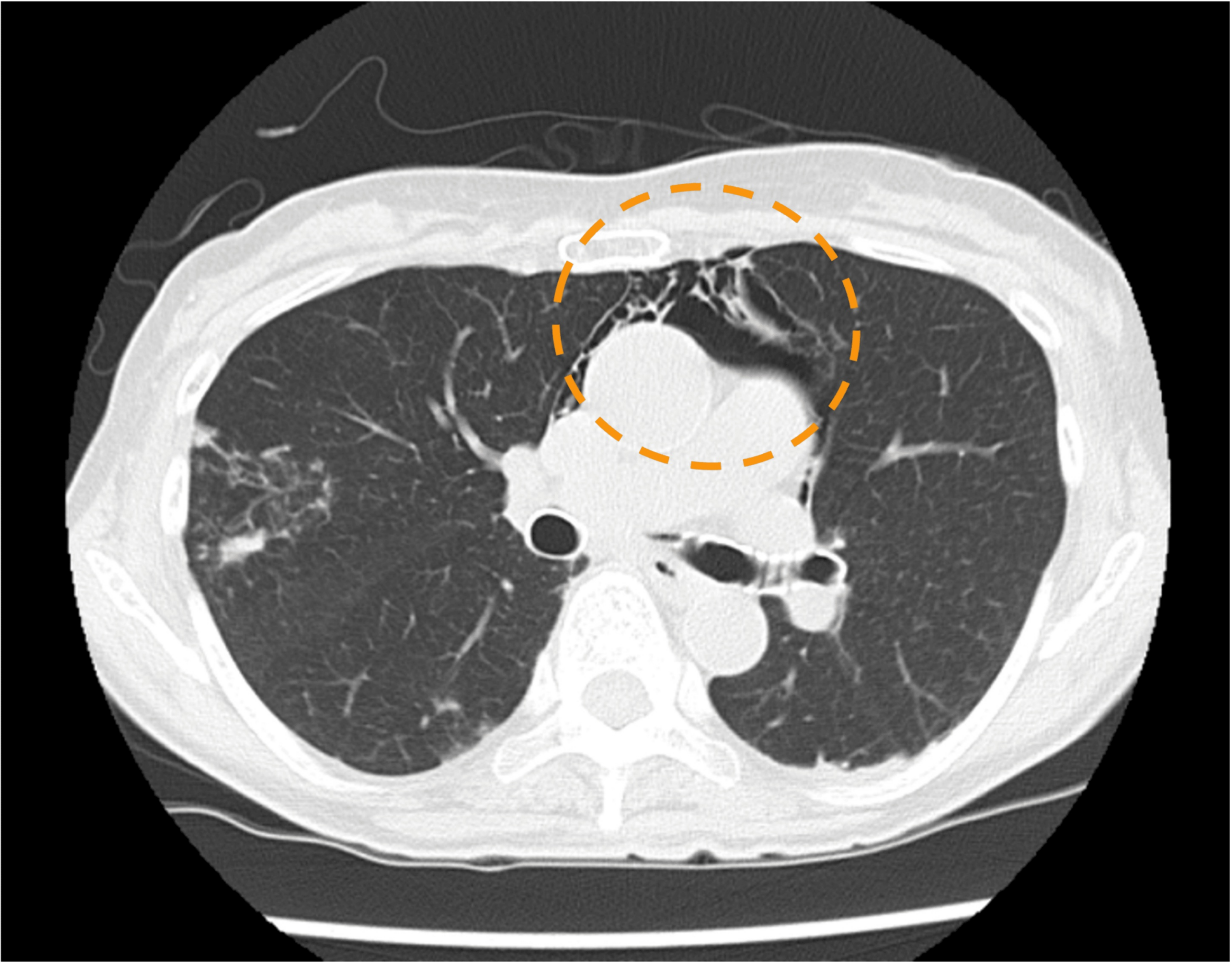
Postoperative CT image showing the presence of free air in the mediastinum and right cervical region CT: computed tomography

**Figure 4:**
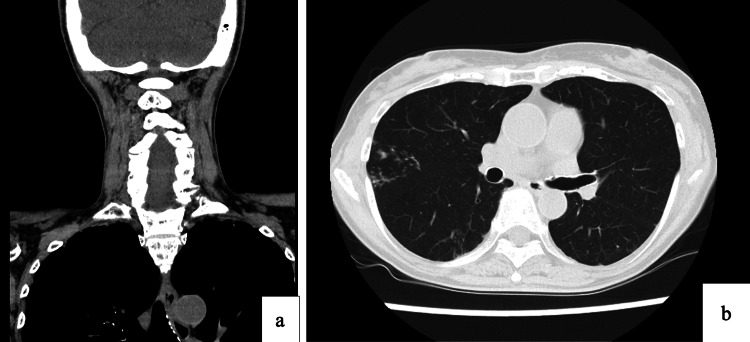
(a, b) CT images on postoperative day 5 confirming the resolution of the gas collections observed immediately post-surgery CT: computed tomography

Immediately post-surgery, cervical and chest radiography and computed tomography (CT) revealed extensive free air tracking from the retropharyngeal space through the right cervical subcutaneous tissues into the mediastinum (Figure 2a-2b and Figure 3). A diagnosis of right cervical SE and PM was established. No evidence of perforation of the digestive tract or trachea was found, and the pharyngeal wall injury during intubation was strongly suspected as the source of the emphysema. To prevent mediastinitis, intravenous administration of ampicillin/sulbactam (6 g/day) was initiated for 48 hours, along with high-concentration oxygen therapy and bed rest. Oxygen therapy was provided at a flow rate of 4 L/min using a face mask. The patient reported chest pain immediately after surgery; however, the symptom resolved spontaneously within 72 hours without hemodynamic or respiratory compromise.　Throughout the clinical course, respiratory and hemodynamic stability were maintained, and no inflammatory response was observed. The patient was discharged on postoperative day 5, after CT confirmed the resolution of the right cervical SE and PM (Figure 4a-4b), and clinical recovery was evident with stable vital signs, no chest symptoms, and tolerance of oral intake.

## Discussion

In the present case, repeated attempts at NTI likely contributed to the laceration of the posterior nasopharyngeal wall, with subsequent positive-pressure ventilation forcing air into the fascial planes, resulting in the spread of emphysema from the cervical region to the mediastinum. Numerous reports have described SE and PM secondary to pharyngeal or tracheal injury during NTI, with repeated attempts and oversized tubes identified as major risk factors [1-3]. The anatomical continuity from the retropharyngeal space through the danger space to the mediastinum has been confirmed in both anatomical and radiological reviews, supporting the plausibility of mediastinal extension [5-7]. Clinically, the most rational diagnostic approach is to suspect SE based on physical findings, such as cervical swelling and crepitus, and evaluate the extent of emphysema and potential complications (e.g., pneumothorax, tracheal or esophageal injury) using CT [4,6].

In most cases, management is conservative, with high-concentration oxygen administration, analgesia, and bed rest as the mainstay [4,8,9]. However, in cases of rapid progression with respiratory or circulatory compromise, prompt surgical interventions, including subcutaneous decompression or mediastinal/thoracic drainage, may be required [4].

Preventive measures include the following: (1) selecting an appropriate tube size and ensuring adequate lubrication during intubation, (2) promptly changing strategies if resistance is encountered, (3) adjusting the bevel or tip orientation, and (4) using a guide when necessary. Tube size must balance nasal passage safety with intratracheal length requirements. Prospective studies have identified sex (for tube diameter) and height (for tube length) as major predictive factors. In addition, partial compression within the nasal cavity or using oversized tubes has been shown to increase airway resistance and predispose to trauma or impaired ventilation [10]. To minimize epistaxis and mucosal injury, randomized controlled trials have demonstrated the benefit of directing the bevel laterally (toward the inferior meatus) or in reverse bevel orientation [11,12]. If resistance occurs, blind repeated attempts should be avoided, and the approach should be promptly switched to orotracheal intubation, video laryngoscopy, or fiberoptic intubation. Furthermore, the use of urinary catheters, suction catheters, or nasopharyngeal airways as guides is useful for securing the passage, particularly in difficult cases or when mucosal injury has already occurred [13-15].

In this case, SE and PM developed in an elderly female patient following multiple NTI attempts, likely facilitated by age-related mucosal vulnerability. Nevertheless, early recognition based on intraoperative findings, followed by CT evaluation of the extent of emphysema, allowed for safe management with supportive therapy and short-term antibiotic administration. In contrast, previously reported severe cases (e.g., extensive SE, PM, and pneumothorax following tracheal rupture after multiple intubation attempts) required invasive drainage or thoracic interventions [2,3]. This underscores the importance of the fundamental principle that states "if resistance is encountered, change the approach".

In oral and maxillofacial surgery under general anesthesia, NTI is often preferred to optimize the surgical access. However, based on the lesion and the instruments required, airway management strategies should be flexibly adapted, including the consideration of orotracheal intubation.

## Conclusions

SE and PM are rare, yet serious, complications that may occur during NTI in oral and maxillofacial surgery. This case highlights that repeated intubation attempts and inappropriate tube size are major risk factors for pharyngeal injury leading to these conditions. Early recognition based on intraoperative findings, prompt imaging evaluation, and conservative management can ensure favorable outcomes. Clinicians should be prepared to alter the airway management strategy when resistance is encountered and consider alternatives, such as orotracheal intubation, for patient safety and an adequate surgical field.
